# Nutritional Habits and Interventions in Childhood

**DOI:** 10.3390/nu14132730

**Published:** 2022-06-30

**Authors:** Silvia Scaglioni, Valentina De Cosmi, Alessandra Mazzocchi

**Affiliations:** 1 Fondazione de Marchi Department of Pediatrics, Fondazione IRCCS Cà Granda Ospedale Maggiore Policlinico, 20122 Milano, Italy; 2 Department of Clinical Sciences and Community Health, University of Milano, 20122 Milano, Italy; valentina.decosmi@gmail.com (V.D.C.); alessandra.mazzocchi1@gmail.com (A.M.)

The present Special Issue of *Nutrients* aims to host scientific articles contributing to enriching the knowledge in the field of nutritional habits and intervention in childhood. The role of the diet in the achievement and maintenance of a healthy status is well recognized. This is especially important in the pediatric age since children need an adequate intake of energy and nutrients for growth and development with respect to their full potential. This collection includes a large group of articles dedicated to the nutrition of the healthy child and its preventive and therapeutic role in numerous diseases (sarcopenia, idiopathic nephrotic syndrome, obesity, anaemia, dyslipidemia, and cardiovascular disease).

Most papers emphasize the importance of establishing proper eating and lifestyle habits to prevent chronic noncommunicable diseases in the first 1000 days and assess different individual and population strategies. Healthy and sustainable food models have been identified: the Mediterranean diet, the new Nordic diet, and the oriental diet, which are the result of ancient traditions but are currently difficult to spread. The main difficulties in identifying effective strategies to improve eating and healthy-living habits are linked to the specificity of the developmental age, the possibility of accessing the various initiatives and, therefore, the socio-economic level, the influence of media and peers, and local traditions. In this editorial, we will provide a look at the topics discussed in each article, providing the novel contributions of the authors on the main theme.

Optimizing nutrition in infancy and establishing healthy lifestyles from preschool years help prevent all forms of malnutrition and diet-related non-communicable diseases in future life. Obesity treatments and preventions in childhood are topics that always offer reasons for study and reflection. In this volume, various therapeutic approaches and suggestions for the treatment and prevention of obesity in the pediatric age are presented and discussed. Alaina P. Vidmar et al. [1] conducted a pilot study on the feasibility and safety of the time-limited eating (TLE) combined with continuous glucose monitoring in 50 adolescents. TLE strategy limits the eating window and may be a feasible approach for treating adolescents with obesity. Calcaterra V. et al. [2], following promising interventions in adults, propose a review of the role of the Ketogenic Diet (KD) as a possible therapeutic tool to counteract metabolic alterations and systemic low-grade inflammation in children and obese adolescents and explain the hypothesized mechanism of action of KD. It is well known that obesity intervention programs should prioritize the low socio-economic families and those with overweight or obese parents. Roberto Franceschi et al. [3] present the “Smuovi La Salute” (“Shake Your Health”) project. This project was targeted to prevent and treat overweight and obesity in low socioeconomic status and minority groups. An app and a cookbook promoting transcultural nutrition and a healthy lifestyle were developed, and no-cost physical activities were organized. Healthy lifestyle teaching was implemented in 30 primary school classrooms. Students’ knowledge of good nutrition significantly improved, and they started to eat more fruits and vegetables. No modifications to the physical activity levels occurred. However, at the individual level, the comprehensive and integrated management of obese patients remains mostly ineffective.

In the context of the prevention of obesity, Kwon et al. [4] described the association between daily milk consumption and the risk of obesity in children aged 30–36 months in Korea. Their data confirmed that participants who consumed an amount higher than the recommendations (more than 500 mL milk per day) were at an increased risk of obesity at the age of 42–72 months, underlining the importance in providing nutritional education to the parents or caregivers. Another study that explored the consumption of milk is the one by Cristine Couto de Almeida et al. [5], which aimed to assess the protein quality and essential amino acid content of both starting and follow-up formulas from different manufacturers in Brazil. The authors found that only some brands exhibited total protein content in accordance with product labels. Protein composition and essential amino acid ratios were variable.

Stival s article [6] is dedicated to both the epidemiological aspect of childhood obesity and overweight in a Nord Italian region and some conditions associated with obesity. In the population examined (2916 adolescents aged 11–15 years), a direct relationship between obesity and increased psychological distress or being victims of bullying has been demonstrated, especially in those with low levels of physical activity. The authors underline the importance of psychological wellbeing since being overweight and having poor physical activity were both related to several shortcomings (i.e., feeling nervous and irritable) and being a victim of bullying. These observations suggest the need for physical activity to always be included in the prevention and treatment of overweight not only for its positive effects on the metabolism but also because participation in sports and physical activity reduces mental health problems developing in adolescence.

Nutritional interventions tailored to specific pathologies are needed to prevent nutritional deficiencies and maintain an adequate nutritional status too. In particular, children and adolescents with chronic or inflammatory diseases are more vulnerable and are at major risks of developing malnutrition. In the presence of cardiovascular risk factors, all guidelines propose dietary behavioural intervention as a first step in treating overweight/obesity. The classification and treatment of an inflammatory condition and metabolic derangement present in numerous pathologies are addressed in some articles in this volume. Giussani et al. designed a study on a cohort of 276 children and adolescents (4–18 years) based on the modification of dietary habits and the general lifestyle, demonstrating an improvement in the weight status and, in most cases, the metabolic alterations (e.g., alterations in the lipid profile, insulin resistance, and hyperuricemia) [7]. In the article by De Cosmi et al., the authors performed a randomized, double-blind, controlled study to evaluate the effects of vitamin D and docosahexaenoic acid (DHA) co-supplementation for six months on vitamin D status, body composition, and metabolic markers of obese children with a vitamin D deficiency. During the supplementation period, all subjects received nutritional advice. At the end of the study, all subjects had fat mass significantly reduced, even if still in a condition of obesity. Children receiving both vitamin D and DHA presented a higher increase in DHA levels that could be relevant to prevent inflammatory-associated complications of obesity, but co-supplementation was no more effective than vitamin D plus placebo [8].

Two articles [9,10] dedicated to cardiovascular diseases and familial hypercholesterolemia underline the role of the preventive and therapeutic intervention of nutrition and discuss dietary recommendations for children [9,10]. In the case of pathology, such as familial hypercholesterolemia, current guidelines support the dietary and lifestyle approach as the primary strategy of intervention in children and adolescents, but additional interventions with nutraceuticals having cholesterol-lowering effects, both as functional foods and as supplements, have been proposed. Banderali et al. in their updated literature review concluded that the use of functional foods as supplements is an interesting strategy for paediatric patients; however, it may have some risks as trials on nutraceuticals have been frequently carried out on a limited study population and the availability of nutraceuticals as supplements without medical prescription could result in uncontrolled use such as auto-prescription, therapy discontinuation, and/or excessive dose, with a consequent reduced therapeutic effect and/or increased adverse events [10]. Capra et al. underline the importance of prudent diet, lifestyle modifications, and the pursuit of psychological wellbeing to prevent the development of dangerous serum lipid levels, excessive adiposity, and elevated blood pressure through intervention in childhood [9].

Rutigliano et al. evaluated the capacity to identify the presence of cardiovascular and metabolic risk of the new guidelines on the diagnosis of hypertension in paediatrics. The authors retrospectively analysed data from 489 overweight and obese children and adolescents [11]. They classified hypertension according to the 2017 American Academy of Pediatrics classification and according to the 2004 classification. The newest ones offer the opportunity to better identify overweight and obese children at risk for organ damage, permitting the design of an earlier prevention strategy. Helgadottir et al. analyzed the macronutrient intake and blood lipid profile at 6 years of age by comparing results from two earlier population-based cohorts of healthy children. They found that a lower intake of saturated and trans-fatty acids, replaced by unsaturated fatty acids, may have contributed to an improved lipid profile in that population. This research work was aimed at the preparation of new national dietary surveys and interventions in childhood [12].

The following articles rely upon nutrition intervention or monitoring and their role in specific disease conditions. Two reviews evaluate the effectiveness of nutritional interventions in improving the quality of the diet of the child population. Qiu et al. analyzed the possible role of the consumption of protein-rich breakfast as a strategy for weight management by declining short-energy intake and suppressing appetite [12]. Most eligible studies were of low quality; therefore, the results ought to be interpreted cautiously. Naroa Andueza et al. systematic reviewed 12 studies and reported that interventions that modify the school environment or provide different meals or snacks may be effective in improving children’s dietary patterns, both in the short and long term [13].

The Developmental Origin of Health and Disease theory, also known as the “Barker hypothesis”, is mentioned in some articles [9,14]. According to Barker’s hypothesis, an individual is programmed toward nutritional thrift during gestation and early postnatal life so that she/he can survive environmental insults caused by poor nutrition. The review by Inzaghi et al. summarized the nutritional roles in the regulation of growth from fetal life to adolescence, attempting to better understand the interplay between nutrients and the endocrine system [14]. The aim was the development of strategies for optimizing nutritional status and allowing the recovery of a normal growth pattern. In this article, the authors recall the fundamental importance of measuring the growth parameters that allow the documentation of regular growth, which is recognized as an excellent and reliable indicator of the child’s general good health. The knowledge of the endogenous and exogenous factors (genetic, endocrine, environmental, nutritional, and socioeconomic) that specifically influence the different growth periods makes it possible to target diagnoses in the case of slowdowns or accelerations with respect to the growth rate.

Achieving an adequate intake of nutrients is an important goal for all ages of life and, particularly, for the pediatric age since it is crucial for cognitive development. Giordani et al. addresses the issue of the Adherence to Dietary Recommendations. The authors used Nutrient Adequacy Ratio (NAR) and Mean Adequacy Ratio (MAR) approaches to evaluate adequacy to Italian dietary reference values at nutrient- and overall-diet levels in 381 seven-year-old children who were previously enrolled within a cohort study aimed at evaluating the effects of mercury on infant neurodevelopment. The study showed a distribution of macronutrient intakes, in the percentage of energy, and it was unbalanced in favour of protein and fats and inadequate with respect to defects for vitamin D, zinc, and folates. Finally, the suboptimal adequacy of the overall diet in the study population emerged [15]. Roberts et al. found significant advances in cognitive outcomes in undernourished preschool-age children who received micronutrient supplementation and improvements in cognitive abilities in nourished children who increase fish consumption [16].

Nutritional status is strictly linked to nutritional habits and, therefore, to nutritional preferences. The cross-sectional study presented by Mumena et al. assessed the role of parents in shaping the dietary behaviours of children. In particular, the impact of maternal knowledge and attitude related to free sugar (FS) was evaluated. Saudi children numbering 424 and aged 6–12 years with their mothers were included, and the results showed that, despite the limited awareness about FS and their influence on health, mothers were making efforts to limit their children’s consumption of this nutrient [17]. Guzek et al. explored the associations between food preferences and appetitive traits in adolescents within the Polish Adolescents’ COVID-19 Experience study population using two validated questionnaires. The results support the association of food preferences with both food approach traits and food avoidance traits [18].

The review from Caffarelli et al. is part of articles that clarify and reinforce EFSA’s messages regarding the timing of egg introduction during complementary feeding. A delayed introduction has no preventive benefit and may negatively influence the growth and psychological wellbeing of children and their families. The authors suggest that HE or HE-containing products should be a regular part of the diet from around 6 months of age, and they should not be introduced earlier than 4 months of age [19].

Vandenplas et al. tested, in a real-life situation, the usefulness of the Cow’s Milk-related Symptom Score (CoMiSS^®^) for the diagnosis and management of infants presenting with symptoms attributable to cow’s milk allergy, suggesting, therefore, that it is an effective tool in aiding the awareness of disease in primary health care [20].

Turolo et al. performed a cross-sectional study to evaluate the positive potential role of the Mediterranean dietary pattern in patients with idiopathic nephrotic syndrome (INS) thanks to their anti-inflammatory properties related to the high levels of omega-3 fatty acids. The authors investigated the adherence to the Mediterranean diet and fatty acid profile and the results in 54 children with Idiopathic Nephrotic Syndrome (INS) and found a negative correlation between proteinuria and the anti-inflammatory omega-3 series [21].

In a cross-sectional study by Sunardi et al., in a sample of 180 children aged 6–36 months living in a poor urban area of Jakarta, results from a nutritional survey detected two dietary determinants as risk factors for anaemia, that there was no cow’s milk formula consumption, and an inadequate zinc intake [22].

The aspect of validated screening tools in paediatrics is intriguing too, in addition to the training of health professionals for their utilization, for preventing both nutritional and psychopathological consequences. The paper by Bertrand et al. reported data about the prevalence of Feeding and Eating Disorders in the general pediatric population, diagnosing the presence of these conditions in at least 12.7% of the children and adolescents [23]. Screening became more than important in the contest of oncologic patients because, in children hospitalized for cancer, malnutrition constitutes a very common complication. Romano et al. examined nutritional and sarcopenia statuses and their clinical impact on pediatric patients’ prognosis affected by bone and soft tissue sarcomas [24].

The message that can arise from all the articles is represented by the fundamental role of the paediatrician as being responsible for health from infancy to adolescence. In particular, the fundamental usefulness of the periodic assessment of nutritional habits and growth parameters, nutritional status, and cognitive development, as part of pediatric check-ups, is examined. Such surveys would allow the early identification of any nutritional errors, eating disorders, and growth alterations and would allow prompt treatment.
